# Microencapsulation of Betaxanthin Pigments from Pitahaya (*Hylocereus megalanthus*) By-Products: Characterization, Food Application, Stability, and *In Vitro* Gastrointestinal Digestion

**DOI:** 10.3390/foods12142700

**Published:** 2023-07-14

**Authors:** María Carolina Otálora, Andrea Wilches-Torres, Jovanny A. Gómez Castaño

**Affiliations:** 1 Grupo de Investigación en Ciencias Básicas (NÚCLEO), Facultad de Ciencias e Ingeniería, Universidad de Boyacá, Tunja 150003, Boyacá, Colombia; andreawilches@uniboyaca.edu.co; 2 Grupo Química-Física Molecular y Modelamiento Computacional (QUIMOL®), Escuela de Ciencias Químicas, Universidad Pedagógica y Tecnológica de Colombia, Sede Tunja 150003, Boyacá, Colombia

**Keywords:** pitahaya peel, betaxanthin, natural dyes, microcapsules, gummy candies, antioxidant capacity, dietary fiber, mucilage

## Abstract

The yellow pitahaya peels generated as by-products during the consumption and processing of the fresh fruit are a rich and underutilized source of betaxanthins (natural yellow-orange pigment with antioxidant activity) and mucilage (structuring material used in the spray-drying process), molecules of high interest for the food industry. In this work, the betaxanthin-rich extract (BRE) obtained from this by-product was microencapsulated by spray drying (SD) using pitahaya peel mucilage (MPP) and maltodextrin (MD) as wall materials. Both types of microencapsulates (i.e., SD-MPP and SD-MD) retained high betaxanthin content (as measured by UV-vis) and antioxidant activity (ORAC). These microencapsulates were characterized structurally (FTIR and zeta potential), morphologically (SEM and particle size/polydispersity index), and thermally (DSC/TGA). The powdered microencapsulates were incorporated into the formulation of candy gummies as a food model, which were subjected to an *in vitro* gastrointestinal digestion process. The characterization study (FTIR and antioxidant activity) of the microcapsules showed that the fruit peel mucilage favors the retention of betaxanthins, while the SEM analysis revealed a particle size of multimodal distribution and heterogeneous morphology. The addition of SD-MPP microcapsules in the candy gummy formulation favored the total dietary fiber content as well as the gumminess and chewiness of the food matrix; however, the inhibition of AAPH^•^ (%) was affected. The stability of the yellow color in the gummies after 30 days of storage indicates its suitability for storage. Consequently, the microencapsulation of betaxanthins with pitahaya peel mucilage can be used as a food additive colorant in the food industry, replacing synthetic colorants, to develop products with beneficial qualities for health that can satisfy the growing demand of consumers.

## 1. Introduction

Natural colors have acquired substantial importance in the food industry. Color is one of the main sensory attributes and quality indicators of a food product, thus influencing acceptability by the consumer. Natural pigments have been widely reported because they maintain sensory quality, enhance the intensity of the original color of the product, and have low toxicity [1].

In recent years, the valorization of fruit processing by-products, such as peels, has drawn the attention of the food industry as a profitable source of natural pigments and mucilage. This new approach arises mainly in response to the growing demand for food products with natural antioxidant attributes and appearance [2], but also due to their mucilaginous content that can be used as a structuring material in technological processes such as spray drying [3].

The pitahaya (*Hylocereus* spp.) peel is a discarded by-product after consumption or processing into juice of the fresh fruit, and still represents 30% of the weight of the whole fruit. This by-product represents an untapped reservoir of bioactive substances, such as betalains, lycopene, polyphenols, flavonoids, carotenoids, phytosterols, dietary fiber, and pectin, which can be used by the food industry as functional natural ingredients in the formulation of new beverages, capsules, and dyes [4,5]. Betalains are natural water-soluble nitrogenous pigments classified into two main chemical families: betacyanins (red-purple substances with *λ_max_*~530 nm) and betaxanthins (yellow-orange substances with *λ_max_*~480 nm). Betalains have demonstrated a versatile application in foods due to their stability range between pH values of 4 to 7 [6] and their broad biological activity, including antioxidant, antitumor, anti-inflammatory, antimicrobial, antiviral, diuretic, antilipidemic, antidiabetic, hepatoprotective, and radioprotective properties [6,7].

In particular, betaxanthins are dyes prone to decomposition due to intrinsic (chemical structure and degrading enzymes) and extrinsic (for example, presence of temperature, water activity, light, oxygen, metal cations, and oxidizing agents) factors, which limits substantially its coloring power, bioavailability (i.e., the percentage of ingested natural pigments that are absorbed by the intestine), and possible application as a functional ingredient in food formulations [6]. In these cases, microencapsulation by atomization has been successfully used in the food industry as a technological strategy for the conservation of coloring and biological properties, as well as a means of transport and distribution of simple bioactive compounds, such as natural pigments. In microencapsulation processes, biopolymers of limited nutritional value such as maltodextrin [8,9] are traditionally used and, more recently, functional biopolymers such as mucilage of cactaceous species [10,11] have been tested. However, the food industry continues to search for new encapsulating materials with functional characteristics, such as intrinsic antioxidant capacity and high dietary fiber content [12,13,14,15]. Recently, it has been shown that mucilages can be used as wall materials in isolation, that is, without the combination with other encapsulating agents, in spray-drying processes [16,17,18]. In this regard, we have recently extracted and characterized the mucilage from the peel of the yellow pitahaya fruit as a wall material for the encapsulation process [19].

The application of microencapsulated orange-yellow pitahaya pigments in food model systems is limited. Among the few studies are the works of Fernandez-López et al. for yogurt and soft drinks [9] and Carmona et al. for yogurt [11].

In the last decade, candy gummies have seen rapid growth in the confectionery industry, attracting the attention of many consumers, from children to the elderly, due to their unique texture, appearance, and taste [20]. Consequently, interest in the fortification of these products has increased considerably during the last decades. However, the applications of betaxanthins in confectionery are limited. In the literature, for example, the addition of betaxanthins from yellow fruits of *Stenocereus pruinosus*, without microencapsulation, is found as one of the few applications in the production of candy gummies [21].

Likewise, information on the release of betaxanthins in the gastrointestinal tract during the digestion of microcapsule-fortified candy gummies is limited. Therefore, it is necessary to know the effect of the microcapsules and the food matrix on the bioaccessibility of this bioactive compound. Ghosh et al. [22] reported that the food matrix could limit the bioaccessibility of betaxanthins, that is, reduce the percentage of ingested natural pigments to be absorbed by the intestine.

The aim of this work was to develop microcapsules containing betaxanthin-rich extract of pitahaya peel using mucilage and maltodextrin as wall materials and incorporate them into gummy candies as a natural colorant to study their AAPH inhibitory activity after *in vitro* ingestion simulation.

## 2. Materials and Methods

### 2.1. Chemicals and Reagents

Maltodextrin (DE-20) was purchased from Cimpa (Bogotá, Colombia). Food grade gelatin was purchased from Gelco (Antioquia, Colombia). Sucrose, glucose syrup, citric acid and 2,2′-Azobis(2-amidinopropane) (AAPH) were supplied by Sigma-Aldrich (St. Louis, MO, USA). Ethanol (analytical grade, 97%) was obtained from Merck (Darmstadt, Germany).

### 2.2. Vegetal Material

Fresh yellow pitahaya peels were collected from local restaurants in the city of Tunja, Boyacá, Colombia. The peels were washed with distilled water at room temperature and cut into small pieces. These pieces were used as a source for extracting both natural pigments (betaxanthins) and mucilage (polysaccharide) of the pitahaya fruit. The mucilage was used as the structuring material for the microencapsulated bioactive compound and the natural pigment as the encapsulated bioactive compound.

### 2.3. Extraction of Betaxanthin Pigment and Mucilage from Pitahaya Peel

The betaxanthin pigment from pitaya fruit peels was extracted using the procedure described by Fathordoobady et al. [23] with some modifications. About 10 g of pitahaya peel pieces was mixed with 300 mL of ethanol/water (50/50 *v*/*v*) solution under constant stirring at 700 rpm at room temperature for 5 h using a magnetic stirrer (C-MAG HS 7 S000, IKA, Staufen Breisgau, Germany). The resulting ethanolic extract was slowly concentrated by drying in an oven at 35 °C for 10 h at atmospheric pressure. The concentrated extract rich in betaxanthins (BRE) was stored in sealed amber bottles at −2 °C until use. The extraction yield of pigment in terms of powder obtained was 1.44 ± 0.51%.

Mucilage extraction was performed following the methodology reported by Otálora et al. [19]. In brief, the fresh and clean fruit peels were cut into small pieces and immersed in 100 mL of distilled water, maintaining a 1:2 (*w*/*v*) ratio for 12 h at room temperature. The plant material was then manually squeezed until a highly viscous gel was extracted. To the pressed gel, 95% ethanol was added in a ratio of 3:1 (ethanol:gel), and the mixture was allowed to stand at room temperature for 15 min without stirring until the formation of a milky white supernatant. This gelatinous supernatant, corresponding to the mucilage, was collected and dried in an oven at 50 °C for 3 h. The dried mucilaginous material was manually macerated until a fine powder was obtained. The extraction yield of mucilage in terms of powder obtained was 1.24 ± 0.50%.

The total phenolic content and antioxidant activity of the powdered mucilage were determined according to the methodology reported by Otálora et al. [3,19].

The powdered mucilage was reconstituted in distilled water, adapting a gel appearance, and was used as wall material in the spray-drying process, as described in Section 2.4.

### 2.4. Spray-Drying Microencapsulation of Pitahaya Peel Betaxanthins

A quantity of 1.0 g of mucilage obtained from yellow pitahaya fruit peels (MPPs) and 1.0 g of maltodextrin DE-20 (MD) were each separately dissolved in 100 mL of distilled water at 18 °C. To ensure complete solubilization, both solutions were constantly stirred at 300 rpm for 6 h and 30 min respectively, using a magnetic stirrer (C-MAG HS 7 S000, IKA, Staufen im Breisgau, Germany). The BRE:wall material ratios in the formulations of the feed mixes were determined on the basis of the literature [16], where the range of total solids content was below 6%, which is suggested as the most suitable for spray drying when only mucilage is used as the wall material.

Two different microcapsule formulations were prepared by dissolving about 2.2 g of BRE in 100 mL mucilage and maltodextrin solutions, respectively. In each case, the mixture was kept under constant stirring for 10 min at 400 rpm, at room temperature, until homogeneous. The total solid content for the mucilage or maltodextrin and betaxanthin extract feed mixture was ≈3.2%. Each feed mixture was then fed into a mini spray dryer (B-290, Büchi Labortechnik, Flawil, Switzerland) using a constant suction of 86% (to maximize the cyclone separation rate) and 40 bar compressed air pressure. The spray-drying process was performed using a nozzle with an internal diameter of 0.7 mm, a feed flow of 350 mL/h, and an inlet air temperature of 170 °C. The two microcapsules obtained i.e., SD-MPP (betaxanthin microcapsules using pitahaya peel mucilage) and SD-MD (betaxanthin microcapsules using maltodextrin) were stored in the dark at room temperature for further analysis and use. The yields of the microencapsulation process (PY) were determined as the ratio of the dry weight of the powder (g) after the spray-drying process to the initial quantity of solids in the feed solution (g) [3].

**Figure 1:**
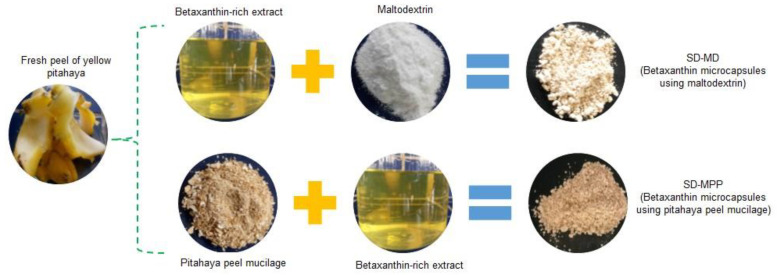
Selected photographs of the microencapsulation process of the extract rich in betaxanthins using pitahaya peel mucilage and maltodextrin as encapsulation materials.

Figure 1 shows photographs of some of the main stages involved in the betaxanthin microencapsulation process using pitahaya peel mucilage or maltodextrin as wall materials.

### 2.5. Physicochemical Characterization of Betaxanthin Microencapsulates

#### 2.5.1. Betaxanthins Content and Antioxidant Activity

The determination of total betaxanthin content was carried out according to the methodology reported by Carmona et al. [11]. Samples of the betaxanthin-rich extract (BRE) and of the SD-MPP and SD-MD microcapsules were each dissolved in a 50:8:42 solution of methanol/acetic acid/water and their absorbance measured at 483 nm in a UV-vis spectrophotometer (V530, Jasco, Hachioji, Tokyo, Japan). The content of betaxanthins (*BC*) expressed in mg/g was determined according to Equation (1).

(1) $$BC=\left(A\times DF\times MW\times Vd\right)/\left(\varepsilon \times L\times Wd\right)$$

where *A* represents the absorption at 483 nm, *DF* is the dilution factor, *Vd* is the volume of the redissolved powder solution (in mL), *Wd* is the weight (in g) of the sample, and *L* is the optical path length (1 cm). *MW* is the molecular weight (350 g/mol) and ε is the molar extinction coefficient at 483 nm (48,000 L/mol cm).

The antioxidant activity was determined by the spectroflurometic method as reported by Otálora et al. [3] using the AAPH radical calculated based on the Trolox calibration curve. Results were expressed as µmol Trolox equivalents/100 g of sample.

#### 2.5.2. Fourier Transform Infrared Spectroscopy (FTIR) and Zeta Potential

The structural properties of the wall materials (i.e., mucilage and maltodextrin), as well as those of SD-MPP and SD-MD microcapsules, were studied by Fourier transform infrared (FTIR) spectroscopy using a Bruker Alpha ECO-ATR spectrophometer (Karlsruhe, Germany) and by zeta potential analysis using a NanoPlus TM 3 Particle Size Zeta Potential Analyzer (Norcross, GA, USA).

#### 2.5.3. Scanning Electron Microscopy and Particle Size/Distribution

The microscopic morphology of SD-MPP and SD-MD microcapsules was examined by scanning electron microscopy (SEM) using an EVO MA 10-Carl Zeiss equipment, Oberkochen, Germany, according to the method described by Otálora et al. [3].

The distribution and size of the particles, as well as the polydispersity index of SD-MPP and SD-MD microcapsules, were determined by laser diffraction using a NanoPlus zeta/nanoparticle analyzer (Micromeritics Instrument CORP, Norcross, GA, USA). The microcapsules were diluted in type I water to adjust the darkening range as follows: viscosity of 0.8878 cP, mean refractive index of 1.33, and sample temperature of 25 °C in a glass cuvette.

#### 2.5.4. Thermal Behavior

The thermal stability of the mucilage used as wall material, as well as that of the SD-MPP and SD-MD microcapsules, was determined by thermogravimetric analysis/differential scanning calorimetry (TGA/DSC) using TA SDT Q600 V20.9 Build 20 instrument (New Castle, DE, USA).

### 2.6. Application of Betaxanthin Microcapsules for Preparation of Gummies

**Figure 2:**
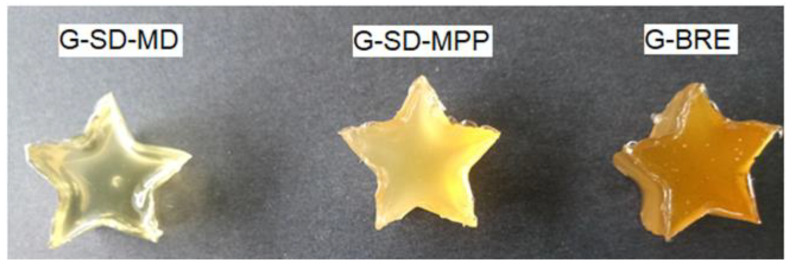
Gummy candies rich in betaxanthins prepared with the addition of SD-MD and SD-MPP microcapsules and BRE extract.

Gummy candies enriched with betaxanthin microcapsules were prepared according to the formulation described by Amjadia et al. [24] with some modifications. Glucose syrup at 31% (*w*/*w*) was mixed with sucrose solution at 34% (*w*/*w*) in distilled water at 60 °C until completely dissolved. To this solution, gelatin was added with a concentration of 8% (*w*/*w*) and the mixture was left under constant stirring for 1 h at 60 °C. Next, 0.3% (*w*/*w*) citric acid was added, and the temperature of the solution was lowered to 40 °C (safe temperature for the addition of microcapsules). Quantities of 2.0% of SD-MPP and SD-MD microcapsules were added separately to produce G-SD-MPP and G-SD-MD food matrices [25]. As a control sample, betaxanthin-enriched gummies were prepared from BRE (G-BRE) using the same procedure described above. The amount of microencapsulated material (SD-MPP and SD-MD) as well as the BRE extract was established in each case in such a way that a color comparable to that of commercial sweet gummies was achieved. The betaxanthin-enriched food matrices (i.e., G-SD-MPP, G-SD-MD, and G-BRE) were poured into a narrow-shaped silicone mold, kept refrigerated for 24 h, then demolded and stored. They were stored at 18 °C until characterization. Figure 2 shows photographs of sweet pitahaya betaxanthin-rich gummies prepared using G-SD-MPP, G-SD-MD, and G-BRE formulations.

### 2.7. Gummy Candy Characterization

#### 2.7.1. Total Dietary Fiber Content

The total dietary fiber content in the G-SD-MPP, G-SD-MD, and G-BRE gummy model samples was determined using a Total Dietary Fiber Test Kit (TDF-100A) provided by Sigma-Aldrich (St. Louis, MO, USA), which is based on the AOAC 985.29 enzymatic-gravimetric method [26].

#### 2.7.2. Texture Analysis

The texture profiles (hardness, stickiness, elasticity, cohesion, gumminess, and resilience) of G-SD-MPP, G-SD-MD and G-BRE samples were determined using a TA-XT plus texture analyzer (Stable Micro Systems, Surrey, UK) and the Exponent software. The betaxanthin-enriched gummies were compressed at room temperature under a cylindrical probe (AB/E-35, diameter 35 mm) at a test speed of 0.5 mm/s and 5.0 mm.

#### 2.7.3. *In Vitro* Evaluation of Gastrointestinal Digestion

The gastrointestinal (GI) digestion profile of the betaxanthins incorporated into the gummy candies (G-SD-MPP, G-SD-MD and G-BRE) was determined by *in vitro* digestion using simulations of the buccal (oral phase during 10 min), stomach (gastric phase for 2 h), and intestinal (intestinal phase for 2 h) media according to the method described by Pacheco et al. [27] with some modifications. Digestive juices were collected after intestinal digestion antioxidant activity analysis following the method described for microcapsules.

Results were expressed as percent-APPH^•^ inhibition calculated using Equation (2).

(2) $$APPH\;inhibition\;[\%]=\left(AO/{AO}_{0}\right)\times 100$$

where AO indicates the ORAC value of nonencapsulated (i.e., G-BRE) or microencapsulated (i.e., G-SD-MPP and G-SD-MD) betaxanthins incorporated into the gummy candies after *in vitro* digestion, and AO_0_ indicates the ORAC value of nonencapsulated (G-BRE) or microencapsulated betaxanthins (G-SD-MPP and G-SD-MD) incorporated into gummy candies before simulated digestion.

#### 2.7.4. Color Stability Analysis

About 1.5 g of each gummy sample (G-SD-MPP, G-SD-MD, and G-BRE) was stored in a desiccator (60 ± 1% relative humidity, RH) containing a saturated sodium bromide solution for 30 days in the absence of light at a constant temperature of 18 ± 1 °C. For each sample, the color attributes (L*, a*, and b*) and the color chroma ($C_{ab}^{*}$) (Equation (3)) and hue ($h_{ab}$) (Equation (4)) parameters were measured at the initial time (zero time) and then after 30 days of storage using a Chroma Meter CR-300 (Konica Minolta Co., Osaka, Japan).

(3) $$C_{ab}^{*}={\left[{\left(a^{*}\right)}^{2}+{\left(b^{*}\right)}^{2}\right]}^{1/2}$$

(4) $$h_{ab}=arctan\left(b^{*}/a^{*}\right)$$

The total color change (ΔE*) for each sample was calculated using Equation (5).

(5) $${∆E}^{*}={\left[{\left(L_{0}^{*}-L_{30}^{*}\right)}^{2}+{\left(a_{0}^{*}-a_{30}^{*}\right)}^{2}+{\left(b_{0}^{*}-b_{30}^{*}\right)}^{2}\right]}^{1/2}$$

where $L_{0}^{*}$, $a_{0}^{*}$, and $b_{0}^{*}$ are the color parameters at zero time and $L_{30}^{*}$, $a_{30}^{*}$, and $b_{30}^{*}$ are the respective values after 30 days of storage.

### 2.8. Statistical Analysis

Betaxanthin content and antioxidant capacity (ORAC), as well as particle size (µm) and zeta potential (mV) data, as presented in Table 1 are reported as the mean ± standard deviation (*n* = 3). Data were analyzed using analysis of variance (ANOVA) and means were compared using Fisher’s least significant difference test (*p* < 0.05).

## 3. Results and Discussion

### 3.1. Spray-Drying Microcapsules’ Characterization

#### 3.1.1. Betaxanthin Content and Antioxidant Capacity

**Figure 3:**
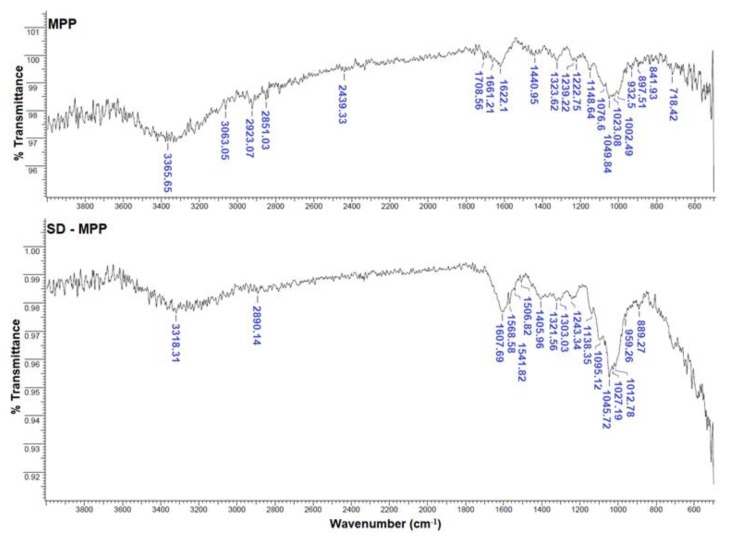
FTIR spectra in the 500–4000 cm^−1^ range of mucilage powder (MPP) samples and betaxanthin SD-MPP microcapsules.

As shown in Table 1, a 17.8% and 19.7% decrease in the betaxanthin content was observed in the SD-MPP and SD-MD microcapsules, respectively, compared to the content originally present in the betaxanthin-rich extract (BRE), which was attributed to lower percentage of betaxanthin in the samples of microcapsules. On the other hand, the higher content of betaxanthins in SD-MPP microcapsules may be related to the high molecular weight of the mucilage polysaccharide, thus allowing an increase in the viscosity of the feed mixture and, therefore, in its emulsifying properties, which contributed to a greater physical stability of the pigment during the spray-drying process [28]. In this way, the mucilage can form a film (i.e., a protective barrier) around the pigment, thus reducing its circulation, deposition, and degradation within the particle surface [29]. In this regard, Antigo et al. [30] observed an increase in betacyanin retention in microcapsules produced with chia mucilage compared to maltodextrin and gum arabic samples. Likewise, the presence of the pigment in the MPP and SD-MD microcapsules was consistent with the results obtained by FTIR (that is, signals observed between 1076 and 889 cm^−1^ assigned to the amino group of the nitrogen (N–H) in the structure of betalains) (see Figure 3a) [31,32].

Likewise, the decrease in the antioxidant activity of the SD-MPP and SD-MD microcapsules compared to that of the betaxanthin-rich extract (BRE) (Table 1) was attributed to the temperature of the spray-drying process, which caused the loss of betaxanthin content, possibly through chemical processes such as isomerization, deglycosylation, and carboxylation reactions [33,34]. The greater antioxidant capacity of SD-MPP microcapsules compared to that of SD-MD microcapsules is due to activity by the mucilage itself, which exerts a protective effect in the betaxanthins, as it is preserved under spray-drying temperatures [35]. In other words, the use of mucilage as wall material in the formulation of SD-MPP microcapsules improved the antioxidant activity by around 42% compared to SD-MD microcapsules formulated with maltodextrin, indicating that the addition of mucilage (activity antioxidant = 1577.64 ± 280.07 µmol Trolox equivalents/g sample on a dry basis) significantly improved the antioxidant activity of betaxanthin microcapsules due to their content of phenolic compounds (25.0-g GAE/100 g sample). The polyphenol content was supported by the presence of a band at 1405 cm^−1^ in the spectra of the SD-MPP microcapsules related to the phenolic–OH groups (see Figure 2) [36].

#### 3.1.2. FTIR Spectra of Microcapsules SD-MPP and SD-MD

**Figure 4:**
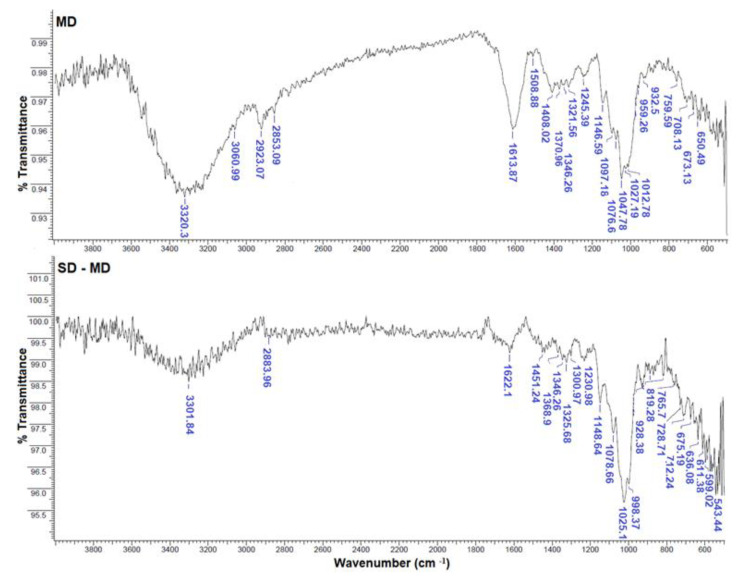
FTIR spectra in the 500–4000 cm^−1^ range of maltodextrin (ED-20) powder (MD) and betaxanthin MD microcapsules (SD-MD).

The FTIR spectra of the wall materials (i.e., mucilage (MPP) and maltodextrin (MD)) and of the microcapsules SD-MPP and SD-MD are presented in Figure 3 and Figure 4.

As shown in Figure 3, the infrared spectrum of the MPP mucilage shows the main bands (that is, the most representative functional groups) of this polymer extracted from the yellow pitaya peel, as we recently reported [19]. When comparing the spectrum of the pitahaya peel mucilage with that of the SD-MPP microcapsules, we observed a displacement in the characteristic bands of the mucilage from 1148.6 and 897.5 cm^−1^ to 1138.3 and 889.2 cm^−1^ respectively, which evidences the formation of electrostatic interactions (that is, hydrogen bonds) [37] between different functional groups of the mucilage [19,38] (anionic polysaccharides [19]) and the functional groups of betalain molecules (anionic form [39] at pH of feed mixtures) [40]. This type of interaction improves pigment retention in the encapsulated matrix as reported in Table 1. The intensity of the band in SD-MPP microcapsules in contrast to MPP evidences the effectiveness of the encapsulation process of the yellow-orange pigment by the mucilage [41]. A similar behavior was reported by Soto-Castro et al. [15] on spray-drying microencapsulation of betalain-rich extracts using cactus mucilage as wall material.

As shown in Figure 4, the infrared spectrum of the ED-20 (MD) maltodextrin material shows characteristic absorption peaks that identify the functional groups of this polymer [42]. Meanwhile, the FTIR spectrum of betaxanthin microcapsules using MD as wall material reveals changes in the wavenumber and relative intensities of maltodextrin peaks from 1077 and 933 cm^−1^ (free sample) to 1095 and 959 cm^−1^, respectively, in SD-MD microcapsules. This shift toward higher frequencies may be associated with the formation of non-covalent interactions, such as hydrogen bonds, between betalain functional groups and the maltodextrin molecule [43]. The similarity of the absorption bands of the SD-MD microcapsules allowed us to confirm the encapsulation of the betalain extract with maltodextrin. A similar behavior was reported by Ferro et al. [44] on the microencapsulation by spray drying of Sida rhombifolia extract using maltodextrin (ED-10) as wall material.

#### 3.1.3. Particle Size, Polydispersity Index, and Zeta Potential of Microcapsules SD-MPP and SD-MD

The distribution and average of the particle size, polydispersity index, and zeta potential of the microcapsules SD-MPP and SD-MD are shown in Table 2.

**Figure 5:**
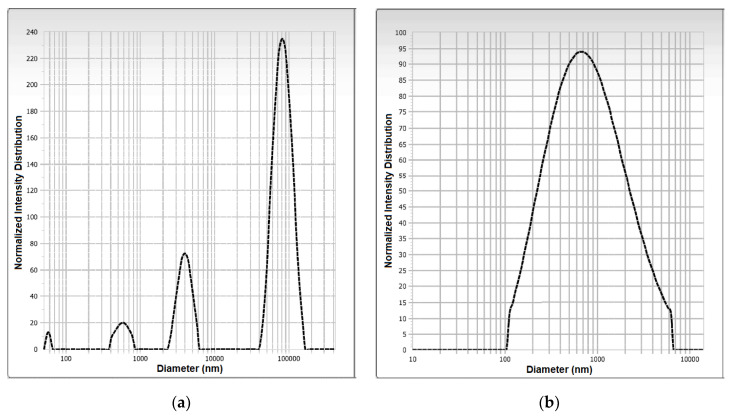
Particle size distribution plots of the SD-MPP (**a**) and SD-MD (**b**) microcapsule samples.

Particle size distribution analysis of microencapsulated samples of pitahaya fruit peel betaxanthins formulated with maltodextrin and mucilage as wall materials exhibited monomodal and multimodal type behavior, respectively (Figure 5a,b). The monomodal behavior (i.e., homogeneous particle size) of SD-MD particles can influence the properties of the powder (i.e., appearance, dispensability, and flowability) and favor its uniform distribution when applied to food, which can in turn affect the texture and sensory characteristics of the food matrix [45]. This type of size distribution was also detected in soybean oil microcapsules produced with maltodextrin as the encapsulating agent [46]. On the other hand, the multimodal distribution in SD-MPP microcapsules indicates a heterogeneous particle size distribution, which would cause uneven dissolution of the powder when applied to a food matrix [47]. A bimodal particle size distribution was observed in gallic acid microcapsules produced with aloe vera mucilage as wall material [12].

As also shown in Table 2, SD-MPP microcapsules showed a higher zeta potential (−30.94 ± 0.74 mW) than SD-MD microcapsules (−25.34 ± 2.60 mW), demonstrating that the presence of mucilage as material wall can promote greater electrical stability than microcapsules formulated with MD [48], because the value of the zeta potential is far from zero [49].

As shown in Figure 5, the average particle size of the microcapsule samples formulated with maltodextrin and mucilage was 1.24 ± 0.16 and 65.01 ± 3.32 µm with polydispersity indices of 0.34 ± 0.00 and 2.58 ± 0.07, respectively. These values allow the powders obtained to be to classified effectively as microcapsules [50] within a desirable size range (i.e., radius < 100 μm), in such a way that they will not affect the sensory characteristics when applied in a food matrix [51]. SD-MD microcapsules show a smaller particle size compared to SD-MPP microcapsules. Consistent with drying theories [14,52], this increase in the particle size of SD-MPP microcapsules may be related to a high viscosity of the feed solution. On the other hand, the decrease in particle size of SD-MD microcapsules can be correlated with the molecular size of maltodextrin (ED-20) and the bulk density of the powder [53]. Similarly, Negrao-Murakami et al. [54] obtained microcapsules of concentrated mate formed with maltodextrin (ED-20) with a particle size of 12.37 ± 1.57 μm.

On the other hand, SD-MPP microcapsules showed larger particles and higher size polydispersity than SD-MD microcapsules. SD-MPP microcapsules with a value greater than 0.5 (2.58 ± 0.07) show a broad size distribution [55] in contrast to SD-MD microcapsules that showed a value less than 0.4 (0.34 ± 0.00), indicating a narrower particle size distribution [56,57].

#### 3.1.4. Microcapsule Morphology by Scanning Electron Microscopy (SEM)

**Figure 6:**
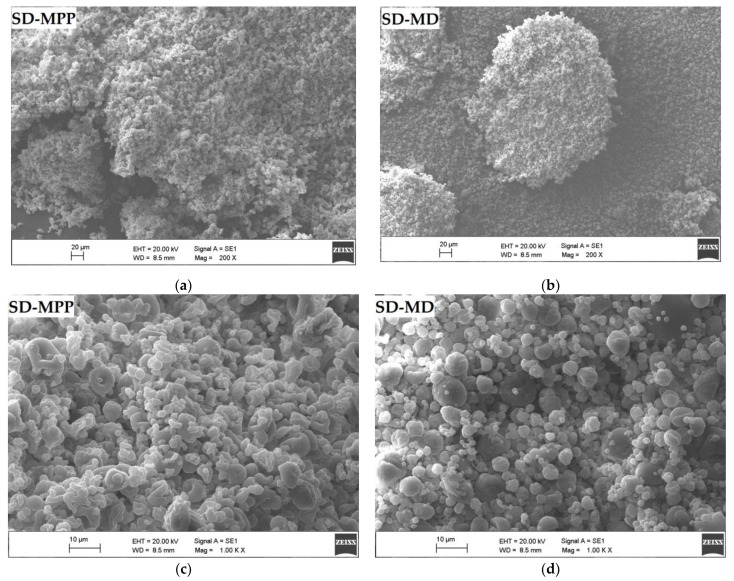

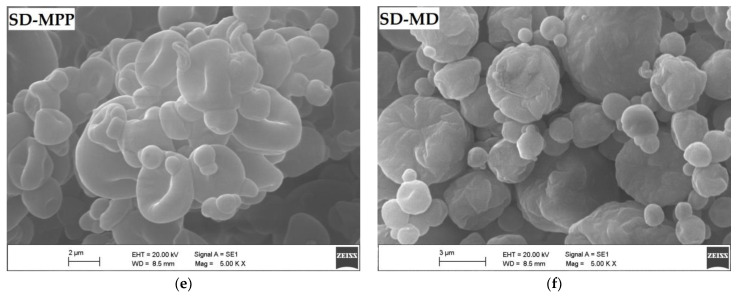
SEM micrographic images of SD-MPP and SD-MD microcapsules taken at magnifications of 200× (**a**,**b**), 1000× (**c**,**d**), and 5000× (**e**,**f**), respectively.

SEM micrographs of SD-MPP and SD-MD microcapsules taken at magnifications of 200×, 1000×, and 5000× are shown in Figure 6, revealing that the morphology of betaxanthin microparticles varies depending on the morphology characteristics of the wall material used [58], that is to say, pitahaya peel mucilage (MPP) [19] and maltodextrin DE-20 [43].

The surface structure of the SD-MPP microcapsules seen at 1000× magnification (Figure 6c) shows a heterogeneous irregular morphology, consistent with the multimodal distribution observed in Figure 5a, with an agglomeration effect between the particles. This could be favorable since it would affect the bulk density of the product, being a quality parameter of the microcapsules [59]. On the other hand, SEM micrographs taken at 5000× magnification (Figure 6e) show how SD-MPP microcapsules exhibit increased particle size compared to SD-MD microcapsules (Figure 6f), which is consistent with the particle size reported in Table 1. At the same magnification, SD-MPP microcapsules are seen as irregular spheres with a smoother and less wrinkled wall compared to SD-MD microcapsules. The above difference is attributed to the presence of mucilage in the feed mixture for SD-MPP, which partially prevents shrinkage, thus influencing the stability of the microencapsulated compound against degrading agents (i.e., oxygen and moisture) and increasing the retention of the bioactive compound (see Table 1), as well as the controlled release [60]. A different morphology was reported by Cortés-Camargo et al. [61] in microencapsulated lemon essential oil using chia mucilage and mesquite gum as wall material.

The SD-MD microcapsules observed at 1000× magnification (Figure 6d) showed a homogeneous morphology with little agglomeration and a monomodal distribution that is consistent with the results obtained in the measurement of the particle size distribution (Figure 5b). The homogeneity of the powder indicates that these spheres could be used in any food product to enhance its coloring properties. As for the SEM micrographs taken at 5000× magnification (Figure 6f), they show how small (radius = 1.24 ± 0.16 µm) SD-MD particles present a dented appearance with a higher degree of wrinkled surfaces than larger SD-MPP particles (radius = 65.01 ± 3.32), possibly due to the contraction of the particles during the drying process, which can be influenced by the high drying speed, the viscosity of the feed mixtures, and the characteristics of the wall material [62]. Janiszewsk [63] reported a similar morphological structure in beetroot juice microcapsules using maltodextrin as encapsulating agent.

#### 3.1.5. Thermal Behavior

**Figure 7:**
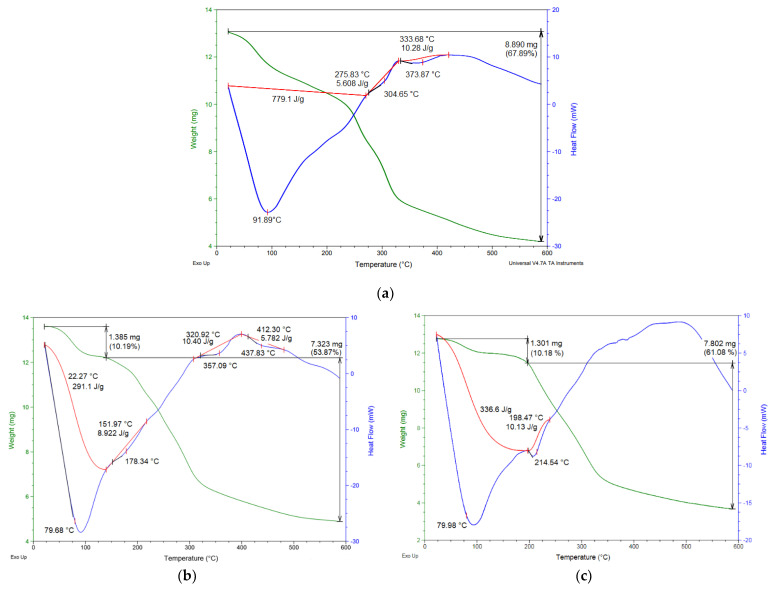
TGA/DSC thermograms of mucilage (**a**), SD-MPP (**b**), and SD-MD (**c**) microcapsules. Heat capacity (J/g °C) (red line).

The thermograms of the wall materials show the typical thermal characteristics reported for this pitahaya peel mucilage [19] (Figure 7a) and for maltodextrin DE-20 [43]. The thermograms of the SD-MPP and SD-MD microcapsules (Figure 7b,c) reveal three main thermal events for both samples divided into two endothermic events and one exothermic event. The first endothermic event occurs between 25 and 200 °C with a weight loss of less than 11% for both samples. This event was associated with the loss of free water (humidity) by evaporation of the powders. The thermograms also show a similarity in the glass transition temperatures (Tg) of 79.68 °C for the SD-MPP microcapsules and 79.98 °C for the SD-MD microcapsules (79.98 °C), which is attributed to the content of sugars (polysaccharides) present in the structure of the wall materials. These Tg values can be attributed to electrostatic interactions or hydrogen bonds between the betaxanthin and the polymers (see Figure 3 and Figure 4). Similar Tg behavior was observed in microencapsulated betalains from *Amaranthus hypochondriacus* using nopal mucilage and maltodextrin (DE-10) as wall materials [18]. These Tg values indicate that both powders should be stored below 80 °C to ensure that the wall materials remain in their amorphous state, thus maintaining the protective barrier around the pigment [64]. The second endothermic event, which occurs at 178.34 °C (SD-MPP powder) and 214.54 °C (SD-MD powder), was associated with the gelatinization process, where starch and carbohydrate chains break down in the presence of water [43]. For its part, the third thermal event in the microcapsules corresponded to an exothermic process that occurred between 400 and 450 °C with a mass loss of 53.87% for the SD-MPP sample and 61.08% for the SD-MD sample. These mass losses were attributed to breakdown/volatilization (i.e., melting) of the polysaccharide backbone of wall materials and possibly partial evaporation of liquids [43]. Similar thermal behavior was observed in the microencapsulation of betaxanthins from *Opuntia megacantha* orange fruits using *Opuntia ficus-indica* cladode mucilage and maltodextrin (DE-20) as wall materials [10].

### 3.2. Characterization of Gummies Formulated with Pitahaya Peel Betaxanthin Microcapsules

Table 3 presents the parameters of the total dietary fiber content (TDFC), texture (hardness, adhesiveness, springiness, cohesiveness, gumminess, and chewiness), and bioaccessibility of the candy gummies produced with the addition of SD-MPP (i.e., G-SD-MPP) and SD-MD (i.e., G-SD-MD) particles, and BRE extract (G-BRE) of betaxanthins extracted from pitahaya peel.

As shown in Table 3, the addition of the SD-MPP microencapsulated in the gummy preparation (sample G-SD-MPP) led to an increase in total dietary fiber content compared to the G-SD-MD and G-BRE candy gummy samples. The origin of the high dietary fiber content in G-SD-MPP gummies can be directly associated with the additional fiber content provided by the mucilage biopolymer (70.51%) [19]. Consequently, the functional properties (i.e., rheological properties) provided by the high dietary fiber content of SD-MPP microcapsules must be considered when they are incorporated as a sustainable colorant-additive during the development of new foods, which would correspond to a possible advantage for the food industry according to the concepts of the circular economy [65]. Compared to the G-SD-MD sample, the G-SD-MPP and G-BRE samples show a higher total dietary fiber content. In particular, the dietary fiber content in the G-BRE sample can be attributed to traces of total fiber present in the peel of the pitahaya fruit (60.11 ± 0.75 [66]). In this way, the formulation of candy gummies (G-SD-MPP) with pitahaya-discard biomass (that is, SD-MPP microcapsules) demonstrates that this by-product can be a sustainable alternative for the food industry that wishes to venture into the formulation of functional products enriched with dragon fruit coloring.

As also shown in Table 2, the gumminess and chewiness of gummy candies prepared with maltodextrin microcapsules (G-SD-MD) were higher compared to those formulated with non-microencapsulated betaxanthins (G-BRE). The increase in these textural properties in the G-SD-MD samples is attributed to the presence of maltodextrin polymers in the formulation of the microcapsules that were incorporated in the preparation of gummies. The presence of these microcapsules in the formulation of sweet gums supports the gel matrix, promoting the union between the gelatin chains to produce defined shapes [25]. On the other hand, the decrease in the gumminess and chewiness values of the gums prepared with G-SD-MPP mucilage microcapsules compared to the G-SD-MD samples may be associated with the disposition and interaction of the mucilage with the ingredients of the formulation of gummy candies [67].

The hardness of the G-SD-MPP and G-SD-MD candy gummies was lower than that of the G-BRE gummies due to the presence of microencapsulates, which can act as humectants and thus retain water within the product (possibly due to the presence of dietary fiber in the mucilage [65]), thus softening the food matrix [68,69]. However, this behavior may also be due to the presence of microencapsulates in the gummy formulation that provided heterogeneity to the network structure by decreasing the number of flexible crosslinks [69]. Likewise, the increase in hardness decreased the elasticity of the G-SD-MPP and G-SD-MD gummy candies [70].

The adhesiveness values of the candy gummies produced with the addition of BRE were lower than those of the candies produced with the SD-MD microcapsules, possibly due to the molecular structure of the product that was affected by the presence of maltodextrin [70]. The incorporation of SD-MPP microcapsules increased the cohesion of the G-SD-MPP sample compared to the G-SD-MD and G-BRE samples. This behavior was attributed to an increase in crosslinks between the gelatin molecules [69], due to the presence of mucilage in the food matrix.

Table 3 further shows the AAPH^•^ inhibition (%) in the different gummy formulations after *in vitro* digestion. The supplementation of candy gummies with microencapsulated SD-MPP and SD-MD provided a higher inhibition of AAPH^•^ (%) compared to the addition of this non-encapsulated bioactive (BRE), which indeed was highly ineffective, demonstrating the impact of microencapsulation in the formulation of this food model. This behavior is because free-form betaxanthins (i.e. unencapsulated betaxanthins) have low solubility and are not resistant to the acidic conditions of the stomach or the alkaline conditions of the intestine [71]. However, the G-SD-MPP matrix showed lower AAPH^•^ (%) inhibition in contrast to the G-SD-MD model food. These results reveal that maltodextrin protects betaxanthins from interactions with other matrix compounds such as proteins present in gelatin, thereby increasing the release of antioxidant molecules from the gummy and, consequently, its solubility [72]. A 135.66% inhibition of DPPH^•^ was observed in soft candies formulated with an emulsion loaded with lutein [67].

On the other hand, the low inhibition of AAPH^•^ (%) in the G-SD-MPP matrix after *in vitro* digestion was possibly because the mucilage, the wall material used in the formulation of microcapsules, under intestine conditions, was not fully hydrolyzed by enzymes during the digestion process, thus slowing the diffusion of betaxanthins [73]. Furthermore, the dietary fibers present in the mucilage can influence the rate of release of antioxidant molecules after digestion because these fibers are digested by bacteria in the colon and not in the upper gastrointestinal tract [74].

### 3.3. Gummy Candies Stability

Table 4 shows the results of the variations in the color values $C_{ab}^{*}$, $h_{ab}$, and ΔE* of the G-SD-MPP, G-SD-MD, and G-BRE gummies after 30 days of storage at 18 °C and 60 ± 1% RH. The parameters $C_{ab}^{*}$ (saturation) and $h_{ab}$ (purity) offer information about the color evolution during storage [75] and, in this case, can be applied to obtain insight about the protective effect of MPP and MD wall materials on stability of betaxanthins in gummy candies. This type of color control during storage has been applied, for example, in yogurt-like fermented soybeans formulated with betalain- and anthocyanin-rich nano-encapsulated and unencapsulated products [76].

After 30 days of storage, the gummies formulated with pitahaya peel mucilage microcapsules (G-SD-MPP) showed an increase of around 31 and 6% in the $C_{ab}^{*}$ and $h_{ab}$ parameters, while the G- SD-MD and G-BRE experienced decreases in the order of 67 and 87% and 57 and 78% in these parameters, respectively. The increase in the parameters $h_{ab}$ (representing the yellow color) and $C_{ab}^{*}$ (representing the strongest color) in the G-SD-MPP sample was attributed to the predominant presence of the light-yellow tone of pitahaya mucilage during the storage time [19], which tends to darken gummy candies. Finally, the total color change (ΔE*) values were lower in G-SD-MPP than in G-SD-MD, indicating that the presence of mucilage improves pigment stability and retards betaxanthin oxidation [77]. For this reason, betaxanthin microencapsulates using mucilage as wall material can be considered as a potential natural yellow dye to be used as a functional additive in the food industry [10].

## 4. Conclusions

In this work, we explored the option of valorizing the peels obtained as by-products of pitahaya fruits by extracting both their mucilage and their pigment (betaxanthins) and using them as the only raw material for the formation of spray-dried microcapsules. For comparative purposes, the physicochemical characterization studies (FTIR, particle size, SEM micromorphology, TGA/DSC, antioxidant capacity, zeta potential), application, and *in vitro* gastric evaluation performed on pitahaya peel fruit microcapsules were also performed on betaxanthin microcapsules using maltodextrin as traditional encapsulating material.

It was shown that the mucilage confers greater protective power to the sensitive pitahaya pigments during the spray-drying process. The greater amount of pigment retained by the mucilage capsules in turn provides a greater antioxidant effect. This result was attributed to the better emulsifying and viscous properties of the mucilage.

On the other hand, the mucilage microcapsules presented a multimodal particle distribution, which differs with the monomodal distribution of the maltodextrin particles. Likewise, the average size and agglomeration capacity of the mucilage particles is greater than that of the particles made with maltodextrin.

Both types of microencapsulated materials showed a similar thermal behavior, featured by two endothermic events and one exothermic event, from which a stable storage temperature below 80 °C was inferred.

The application of the microencapsulated materials consisted of their addition to a sweet food matrix in the form of gummies, where a gummy formulation with non-encapsulated betaxanthin extract was established as a control sample. The candy gummies enriched with mucilage microcapsules had a higher content of dietary fiber, with a lower degree of gumminess and chewiness, than the maltodextrin gummies and the control sample. These model sweets were additionally used for *in vitro* digestibility tests, from which it was possible to demonstrate a greater AAPH^•^ inhibition effect for the candy formulation using maltodextrin. Finally, the color stability test of the gummy samples for 30 days of storage at 18 °C and 60 ± 1% RH revealed a superior protective effect of the betaxanthin pigments in the case of the formulation with pitahaya peel mucilage microcapsules. In summary, this work demonstrates the underutilized potential of pitahaya fruit by-product pigments as a source of 100% natural colorants for the formulation of functional microencapsulates for the food industry.

## 5. Patents

The results of this work are a structural part of the National Invention Patent Application No. NC2022/0007738 submitted for evaluation to the Superintendencia de Industria y Comercio of Colombia.

## Figures and Tables

**Table 1:** Process yield (PY), total content of betaxanthins (BC), and antioxidant capacity of the extract (BRE) and of the microcapsules (SD-MPP and SD-MD) that contain pigment extracted from yellow pitaya peel.

Parameter	BRE	SD-MPP	SD-MD
PY (%)		25.2	50.4
BC ^1^	0.1058	0.087	0.085
ORAC ^2^	1298.49 ± 160.82 ^a^	868.68 ± 114.23 ^b^	366.59 ± 47.31 ^c^

^1^ BC is represented as mg betaxanthins/g of sample. ^2^ ORAC is represented as µmol Trolox equivalents/g of samples in dry base. Different letters in the same row and column for each parameter indicate a statistical difference (*p* < 0.05) between samples.

**Table 2:** Particle size (PS), polydispersity index (PDI), and zeta potential parameters of the microcapsules SD-MPP and SD-MD that contain pigment extracted from yellow pitaya peel.

Parameter ^1^	SD-MPP	SD-MD
PS (in µm)	65.01 ± 3.32 ^a^	1.24 ± 0.16 ^b^
PDI	2.58 ± 0.07 ^a^	0.34 ± 0.00 ^b^
Zeta potential	−30.94 ± 0.74 ^a^	−25.34 ± 2.60 ^b^

^1^ Different letter in the same row and column for each parameter indicate a statistical difference (*p* < 0.05) between samples.

**Table 3:** Total dietary fiber content (TDFC), texture, and bioaccessibility values of candy gummies produced with the addition of SD-MPP and SD-MD microcapsules, and betaxanthin-rich extract (BRE).

Parameter	G-SD-MPP	G-SD-MD	G-BRE
TDFC ^1^	0.82	0.26	0.56
Hardness (g)	177.3	346.6	531.6
Adhesiveness (g·s)	-	−101.1	−19.8
Springiness (mm)	0.970	0.970	0.908
Cohesiveness (-)	450.9	0.914	0.661
Gumminess (g)	151.0	310.8	268.9
Chewiness (g)	147.1	299.6	233.5
AAPH^•^ Inhibition (%) ^2^	347.3	528.2	155.1

^1^ TDFC is expressed as g/100 g. ^2^ Expressed as ORAC (µmol Trolox equivalents/g of sample in dry base).

**Table 4:** Evolution of chroma ($C_{ab}^{*}$ ) and hue angle ($h_{ab}$ ) parameters of gummy candies (G-SD-MPP, G-SD-MD and G-BRE) during storage at 0 and 30 days.

Sample ^1^	Time (in Days)	$C_{ab}^{*}$	$h_{ab}$	ΔE*
G-SD-MPP	0	531.20 ± 73.02 ^c^	76.20 ± 1.54 ^b^	9.94 ± 2.07 ^c^
	30	770.27 ± 21.71 ^a^	80.68 ± 0.14 ^a^	
G-SD-MD	0	343.05 ± 9.36 ^d^	82.58 ± 0.26 ^a^	10.69 ± 1.03 ^b^
	30	115.01 ± 22.41 ^f^	10.69 ± 1.03 ^d^	
G-BRE	0	676.51 ± 49.48 ^b^	76.70 ± 0.47 ^b^	17.05 ± 0.72 ^a^
	30	291.43 ± 24.81 ^e^	17.05 ± 0.72 ^c^	

^1^ Different letters in the same column for each color parameter indicate a statistical difference (*p* < 0.05) between samples.

## Data Availability

The data presented in this study are available on request from the corresponding author.

## References

[1] Echegaray N., Guzel N., Kumar M., Guzel M., Hassoun A., Lorenzo J.M. (2023). Recent advancements in natural colorants and their application as coloring in food and in intelligent food packaging. Food Chem..

[2] Cassani L., Marcovich N.E., Gomez-Zavaglia A. (2022). Valorization of fruit and vegetables agro-wastes for the sustainable production of carotenoid-based colorants with enhanced bioavailability. Food Res. Int..

[3] Otálora M.C., Wilches-Torres A., Gómez Castaño J.A. (2023). Spray-Drying. Microencapsulation of Andean Blueberry (Vaccinium meridionale Sw.) Anthocyanins Using Prickly Pear (*Opuntia ficus indica* L.) Peel Mucilage or Gum Arabic: A Comparative Study. Foods.

[4] Jiang H., Zhang W., Pu Y., Chen L., Cao J., Jiang W. (2023). Development and characterization of a novel active and intelligent film based on pectin and betacyanins from peel waste of pitaya (*Hylocereus undatus*). Food Chem..

[5] Jiménez-Aguilar D.M., López-Martínez J.M., Hernández-Brenes C., Gutiérrez-Uribe J.A., Welti-Chanes J. (2015). Dietary fiber, phytochemical composition and antioxidant activity of Mexican commercial varieties of cactus pear. J. Food Compost. Anal..

[6] Carreon-Hidalgo J.P., Franco-Vasquez D.C., Gomez-Linton D.R., Perez-Flores L.J. (2022). Betalain plant sources, biosynthesis, extraction, stability enhancement methods, bioactivity, and applications. Food Res. Int..

[7] Rodríguez-Mena A., Ochoa-Martínez L.A., Gonzalez-Herrera S.M., Rutiaga-Quinones O.M., Gonzalez-Laredo R.F., Olmedilla-Alonso B. (2023). Natural pigments of plant origin: Classification, extraction and application in foods. Food Chem..

[8] Gandía-Herrero F., Jimenez-Atienzar M., Cabanes J., García-Carmona F., Escribano J. (2010). Stabilization of the bioactive pigment of Opuntia fruits through maltodextrin encapsulation. J. Agric. Food Chem..

[9] Fernandez-Lopez J.A., Roca M.J., Angosto J.M., Obon J.M. (2018). Betaxanthin-rich extract from cactus pear fruits as yellow water-soluble colorant with potential application in foods. Plant Foods Hum. Nutr..

[10] Otalora M.C., Carriazo J.G., Osorio C., Nazareno M.A. (2018). Encapsulation of cactus (*Opuntia megacantha*) betaxanthins by ionic gelation and spray drying: A comparative study. Food Res. Int..

[11] Carmona J.C., Robert P., Vergara C., Saenz C. (2021). Microparticles of yellow-orange cactus pear pulp (*Opuntia ficus-indica*) with cladode mucilage and maltodextrin as a food coloring in yogurt. LWT Food Sci. Technol..

[12] Medina-Torres L., Núñez-Ramírez D.M., Calderas F., González-Laredo R.F., Minjares-Fuentes R., Valadez-García M.A., Bernad-Bernad M.J., Manero O. (2019). Microencapsulation of gallic acid by spray drying with aloe vera mucilage (*Aloe barbadensis miller*) as wall material. Ind. Crop. Prod..

[13] Otálora M.C., Wilches-Torres A., Gómez Castaño J.A. (2021). Extraction and Physicochemical Characterization of Dried Powder Mucilage from Opuntia ficus-indica Cladodes and Aloe Vera Leaves: A Comparative Study. Polymers.

[14] De Campo C., Dick M., dos Santos P.P., Costa T.M.H., Paese K., Guterres S.S., Rios A.O., Flôres S.H. (2018). Zeaxanthin nanoencapsulation with Opuntia monacantha mucilage as structuring material: Characterization and stability evaluation under different temperatures. Colloids Surf. A Physicochem. Eng. Asp..

[15] Soto-Castro D., Gutiérrez M.G., León-Martínez F., Santiago-García P.A., Aragón-Lucero I., Antonio-Antonio F. (2019). Spray drying microencapsulation of betalain rich extracts from Escontria chiotilla and Stenocereus queretaroensis fruits using cactus mucilage. Food Chem..

[16] Otálora M.C., Wilches-Torres A., Gómez Castaño J.A. (2022). Spray-Drying Microencapsulation of Pink Guava (*Psidium guajava*) Carotenoids Using Mucilage from *Opuntia ficus-indica* Cladodes and Aloe Vera Leaves as Encapsulating Materials. Polymers.

[17] Medina-Torres L., García-Cruz E.E., Calderas F., González Laredo R.F., Sánchez-Olivares G., Gallegos-Infante J.A., Rocha-Guzmán N.E., Rodríguez-Ramírez J. (2013). Microencapsulation by spray drying of gallic acid with nopal mucilage (*Opuntia ficus indica*). LWT—Food Sci. Technol..

[18] Tabio-García D., Paraguay-Delgado F., Lardizabal Gutiérrez D., Quintero-Ramos A., Meléndez-Pizarro C.O., Ochoa-Martínez L.A., Sánchez-Madrigal M.A., Ruiz-Gutiérrez M.G., Espinoza-Hicks J.C. (2023). Effectiveness of *Opuntia ficus-indica* mucilage as a carrier agent in microencapsulation of bioactive compounds of Amaranthus hypochondriacus var. Nutrisol. Food Biosci..

[19] Otálora M.C., Wilches-Torres A., Gómez Castaño J.A. (2023). Mucilage from Yellow Pitahaya (*Selenicereus megalanthus*) Fruit Peel: Extraction, Proximal Analysis, and Molecular Characterization. Molecules.

[20] Gunes R., Palabiyik I., Konar N., Said Toker O. (2022). Soft confectionery products: Quality parameters, interactions with processing and ingredients. Food Chem..

[21] Rodríguez-Sanchez J.A., Cruz y Victoria M.T., Barragan-Huerta B.E. (2017). Betaxanthins and antioxidant capacity in Stenocereus pruinosus: Stability and use in food. Food Res. Int..

[22] Ghosh S., Sarkar T., Das A., Chakraborty R. (2022). Natural colorants from plant pigments and their encapsulation: An emerging window for the food industry. LWT—Food Sci. Technol..

[23] Fathordoobady F., Mirhosseini H., Salamat J., Abd Manap M.Y. (2016). Effect of Solvent Type and Ratio on Betacyanins and Antioxidant Activity of Extracts from Hylocereus polyrhizus Flesh and Peel by Supercritical Fluid Extraction and Solvent Extraction. Food Chem..

[24] Amjadia S., Ghorbani M., Hamed Hamishehkar H., Roufegarinejad L. (2018). Improvement in the stability of betanin by liposomal nanocarriers: Its application in gummy candy as a food model. Food Chem..

[25] Charoen R. (2015). Development of Antioxidant Gummy Jelly Candy Supplemented with Psidium guajava Leaf Extract. Int. J. Appl. Sci. Technol..

[26] Cunniff P. (1997). Enzymatic-gravimetric method. Official Methods of Analysis of AOAC International.

[27] Pacheco C., González E., Robert P., Parada J. (2018). Retention and pre-colon bioaccessibility of oleuropein in starchy food matrices, and the effect of microencapsulation by using inulin. J. Funct. Foods.

[28] Us-Medina U., Julio L.M., Segura-Campos M.R., Ixtaina V.Y., Tomas M.C. (2018). Development and characterization of spray-dried chia oil microcapsules using by-products from chia as wall material. Powder Technol..

[29] de Freitas Santos P., Rubio Vieira F.T., Palazzolli da Silva M., Siva Pinho L., Favaro-Trindade C.S. (2021). Microencapsulation of carotenoid-rich materials: A review. Food Res. Int..

[30] Antigo J.L.D., Stafussa A.P., de Cassia Bergamasco R., Madrona G.S. (2020). Chia seed mucilage as a potential encapsulating agent of a natural food dye. J. Food Eng..

[31] Halloub A., Raji M., Essabir H., Nekhlaoui S., Bensalah M.-O., Bouhfid R., el kacem Qaiss A. (2023). Stable smart packaging betalain-based from red prickly pear covalently linked into cellulose/alginate blend films. Int. J. Biol. Macromol..

[32] Calva-Estrada S.J., Jiménez-Fernández M., Lugo-Cervantes E. (2022). Betalains and their applications in food: The current state of processing, stability and future opportunities in the industry. Food Chem. Mol. Sci..

[33] Azeredo H.M.C. (2009). Betalains: Properties, sources, applications, and stability—A review. Int. J. Food Sci. Technol..

[34] Herbach K.M., Stintzing F.C., Carle R. (2006). Betalain stability and degradationstructural and chromatic aspects. J. Food Sci..

[35] Medina-Torres L., Núñez-Ramírez D.M., Calderas F., Bernad-Bernad M.J., Gracia-Mora J., Rodríguez-Ramírez J., González-Laredo R.F., Gallegos-Infante J.A., Manero O. (2018). Curcumin encapsulation by spray drying using Aloe vera mucilage as encapsulating agent. J. Food Process Eng..

[36] Gheribi R., Habibi Y., Khwaldia K. (2019). Prickly pear peels as a valuable resource of added-value polysaccharide: Study of structural, functional and film forming properties. Int. J. Biol. Macromol..

[37] Utpott M., Queiroz Assis R., Pagno C.H., Pereira Krigger S., Rodrigues E., de Oliveira Rios A., Flôres S.H. (2020). Evaluation of the Use of Industrial Wastes on the Encapsulation of Betalains Extracted from Red Pitaya Pulp (Hylocereus polyrhizus) by Spray Drying: Powder Stability and Application. Food Bioprocess Technol..

[38] Bayar N., Kriaa M., Kammoun R. (2016). Extraction and characterization of three polysaccharides extracted from *Opuntia ficus indica* cladodes. Int. J. Biol. Macromol..

[39] Miguel M.G. (2018). Betalains in some species of the amaranthaceae family: A review. Antioxidants.

[40] Robert P., Torres V., García P., Vergara C., Saenz C. (2015). The encapsulation of purple cactus pear (*Opuntia ficus-indica*) pulp by using polysaccharide-proteins as encapsulating agents. LWT—Food Sci. Technol..

[41] Monge Neto A.A., Fonseca Tomazini L., Gouveia Mizuta A., Gomes Correa R.C., Scaramal Madrona G., Faria de Moraes F., Peralta R.M. (2021). Direct microencapsulation of an annatto extract by precipitation of psyllium husk mucilage polysaccharides. Food Hydrocoll..

[42] Alves E.S., Ripke Ferreira C.S., Souza P.R., Silva Bruni A.R., Campos Castro M., Bruno Figueiredo Saqueti H., Oliveira Santos O., Scaramal Madrona G., Vergilio Visentainer J. (2023). Freeze-dried human milk microcapsules using gum arabic and maltodextrin: An approach to improving solubility. Int. J. Biol. Macromol..

[43] Otálora M.C., Carriazo J.G., Iturriaga L., Nazareno M.A., Osorio C. (2015). Microencapsulation of betalains obtained from cactus fruit (*Opuntia ficus-indica*) by spray drying using cactus cladode mucilage and maltodextrin as encapsulating agents. Food Chem..

[44] Ferro D.M., Oliveira Müller C.M., Salvador Ferreira S.R. (2020). Photostability and characterization of spray-dried maltodextrin powders loaded with Sida rhombifolia extract. Biocatal. Agric. Biotechnol..

[45] Fernandes R.V., Borges S.V., Botrel D.A. (2014). Gum Arabic/starch/maltodextrin/inulin as wall materials on the microencapsulation of rosemary essential oil. Carbohydr. Polym..

[46] Zhu J., Li X., Liu L., Li Y., Qi B., Jiang L. (2020). Preparation of spray-dried soybean oil body microcapsules using maltodextrin: Effects of dextrose equivalence. LWT—Food Sci. Technol..

[47] Santiago-Adame R., Medina-Torres L., Gallegos-Infante J.A., Calderas F., González-Laredo R.F., Rocha-Guzmán N.E., Ochoa-Martínez L.A., Bernad-Bernad M.J. (2015). Spray drying-microencapsulation of cinnamon infusions (Cinnamomum zeylanicum) with maltodextrin. LWT—Food Sci. Technol..

[48] Li X., Zhang Z.-H., Qiao J., Qu W., Wang M.-S., Gao X., Zhang C., Brennan C.S., Qi X. (2022). Improvement of betalains stability extracted from red dragon fruit peel by ultrasound-assisted microencapsulation with maltodextrin. Ultrason. Sonochem..

[49] Lee Y.-K., Chang Y.H. (2020). Microencapsulation of a maca leaf polyphenol extract in mixture of maltodextrin and neutral polysaccharides extracted from maca roots. Int. J. Biol. Macromol..

[50] Kwak H.-S., Al Mijan M., Ganesan P. (2014). Application of nanomaterials, nano-and microencapsulation to milk and dairy products. Nano- Microencapsul. Foods.

[51] Burgain J., Gaiani C., Linder M., Scher J. (2011). Encapsulation of probiotic living cells: From laboratory scale to industrial applications. J. Food Eng..

[52] Muzaffar K., Kumar P. (2015). Parameter optimization for spray drying of tamarind pulp using response surface methodology. Powder Technol..

[53] Tonon R.V., Brabet C., Hubinger M.D. (2010). Anthocyanin stability and antioxidant activity of spray-dried açai (Euterpe oleracea Mart.) juice produced with different carrier agents. Food Res. Int..

[54] Negrao-Murakami A.N., Nunes G.L., Pinto S.S., Murakami F.S., Amante E.R., Cunha Petrus J.C., Prudencio E.S., Amboni R.D.M.C. (2017). Influence of DE-value of maltodextrin on the physicochemical properties, antioxidant activity, and storage stability of spray dried concentrated mate (Ilex paraguariensis A. St. Hil.). LWT—Food Sci. Technol..

[55] Molaveisi M., Noghabi M.S., Parastouei K., Taheri R.A. (2021). Fate of nanophytosomes containing bioactive compounds of Echinacea extract in an acidic food beverage. Food Struct..

[56] Parvez S., Wani I.A., Masoodi F.A. (2022). Nanoencapsulation of green tea extract using maltodextrin and its characterisation. Food Chem..

[57] Jang Y., Koh E. (2023). Characterisation and storage stability of aronia anthocyanins encapsulated with combinations of maltodextrin with carboxymethyl cellulose, gum Arabic, and xanthan gum. Food Chem..

[58] Aberkane L., Roudaut G., Saurel R. (2014). Encapsulation and oxidative stability of PUFA-rich oil microencapsulated by spray drying using pea protein and pectin. Food Bioprocess Technol..

[59] Mestry A.P., Mujumdar A.S., Thorat B.N. (2011). Optimization of spray drying of an innovative functional food: Fermented mixed juice of carrot and watermelon. Dry Technol..

[60] Osorio C., Acevedo B., Hillebrand S., Carriazo J., Winterhalter P., Morales A.L. (2010). Microencapsulation by spray-drying of anthocyanin pigments from Corozo (*Bactris guineensis*) fruit. J. Agric. Food Chem..

[61] Cortes-Camargo S., Acuña-Avila P.E., Rodriguez-Huezo M.E., Roman-Guerrero A., Varela-Guerrero V., Perez-Alonso C. (2019). Effect of chia mucilage addition on oxidation and release kinetics of lemon essential oil microencapsulated using mesquite gum—Chia mucilage mixtures. Food Res. Int..

[62] Jafari S.M., Assadpoor E., He Y., Bhandari B. (2008). Encapsulation efficiency of food flavours and oils during spray drying. Dry. Technol..

[63] Janiszewsk E. (2014). Microencapsulated beetroot juice as a potential source of betalain. Powder Technol..

[64] Risch S.J., Reineccius G.A. (1995). Encapsulation and controlled release of food ingredients. ACS symposium Series.

[65] Carpena M., Cassani L., Gomez-Zavaglia A., Garcia-Perez P., Seyyedi-Mansour S., Cao H., Simal-Gandara J., Prieto M.A. (2023). Application of fermentation for the valorization of residues from Cactaceae family. Food Chem..

[66] Montoya-Arroyo A., Schweiggert R.M., Pineda-Castro M.L., Sramek M., Kohlus R., Carle P., Esquivel R. (2014). Characterization of cell wall polysaccharides of purple pitaya (*Hylocereus* sp.) pericarp. Food Hydrocoll..

[67] Pan L.-H., Wu C.-L., Luo S.-Z., Luo J.-P., Zheng Z., Jiang S.-T., Zhao Y.-Y., Zhong X.-Y. (2022). Preparation and characteristics of sucrose-resistant emulsions and their application in soft candies with low sugar and high lutein contents and strong antioxidant activity. Food Hydrocoll..

[68] Constantino A.B.T., Garcia-Rojas E.E. (2023). Microencapsulation of beta-carotene by complex coacervation using amaranth carboxymethyl starch and lactoferrin for application in gummy candies. Food Hydrocoll..

[69] Hani N.M., Romli S.R., Ahmad M. (2015). Influences of red pitaya fruit puree and gelling agents on the physico-mechanical properties and quality changes of gummy confections. Int. J. Food Sci. Technol..

[70] Mutlua C., Tontula S.A., Erbaşa M. (2018). Production of a minimally processed jelly candy for children using honey instead of sugar. LWT—Food Sci. Technol..

[71] Fredes C., Osorio M.J., Parada J., Robert P. (2018). Stability and bioaccessibility of anthocyanins from maqui (Aristotelia chilensis [Mol.] Stuntz) juice microparticles. LWT—Food Sci. Technol..

[72] Grenha A., Guerreiro F., Lourenço J.P., Lopes J.A., Cámara-Martos F. (2023). Microencapsulation of selenium by spray-drying as a tool to improve bioaccessibility in food matrix. Food Chem..

[73] Cakmak H., Ilyasoglu-Buyukkestelli H., Sogut E., Hazal Ozyurt V., Gumus-Bonacina C.E., Sebnem Simsek S. (2023). A review on recent advances of plant mucilages and their applications in food industry: Extraction, functional properties and health benefits. Food Hydrocoll. Health.

[74] Zhang Z., Zhang R., Zou L., Chen L., Ahmed Y., Al Bishri W., McClements D.J. (2016). Encapsulation of curcumin in polysaccharide-based hydrogel beads: Impact of bead type on lipid digestion and curcumin bioaccessibility. Food Hydrocoll..

[75] Kaimainen M., Laaksonen O., Järvenpää E., Sandell M., Huopalahti R. (2015). Consumer acceptance and stability of spray dried betanin in model juices. Food Chem..

[76] Dias S., Castanheira E.M.S., Fortes A.G., Pereira D.M., Gonçalves M.S.T. (2020). Natural pigments of anthocyanin and betalain for coloring soy-based yogurt alternative. Foods.

[77] Khan M.I. (2016). Stabilization of betalains: A review. Food Chem..

